# Structure and Function Analysis of Cultivated *Meconopsis integrifolia* Soil Microbial Community Based on High-Throughput Sequencing and Culturability

**DOI:** 10.3390/biology12020160

**Published:** 2023-01-19

**Authors:** Yan Wang, Qingyun Ma, Lingling Wang, Jingkuo Hu, Huiying Xue, Dongfei Han, Zhen Xing, Zhiyong Ruan

**Affiliations:** 1 College of Resources and Environment, Tibet Agricultural and Animal Husbandry University, Linzhi 860000, China; 2 Institute of Agricultural Resources and Regional Planning, Chinese Academy of Agricultural Sciences, Beijing 100081, China; 3 State Key Laboratory of Agricultural Microbiology, Huazhong Agricultural University, Wuhan 430070, China; 4 School of Environmental Science and Engineering, Suzhou University of Science and Technology, Suzhou 215011, China; 5 College of Life Sciences, Yantai University, Yantai 264005, China

**Keywords:** *Meconopsis integrifolia*, soil microorganism, high-throughput sequencing, cultivable method

## Abstract

**Simple Summary:**

The composition, function, and interactions of the soil microbial community of cultivated *Meconopsis integrifolia* are characterized by high-throughput sequencing technology and culturing methods. Soil bacteria are mainly involved in nutrient cycling, and mycorrhizal fungi form symbioses with plants. Both bacteria and fungi adopt a more synergistic and cooperative-oriented strategy to promote overall metabolic efficiency toward maintaining survival in extreme habitats and at the expense of reduced ecological stability. The results of this study offer great guidance for the conservation of endangered wild resources of *Meconopsis integrifolia* and the exploitation of its medicinal value.

**Abstract:**

(1) Background: The structure, function, and community interactions of soil microbial communities of cultivated *Meconopsis integrifolia* were characterized by studying this alpine flower and traditional endangered Tibetan medicine. (2) Methods: Soil bacteria and fungi were studied based on high-throughput sequencing technology. Bacteria were isolated using culturomics and functionally identified as IAA-producing, organic phosphorus-dissolving, inorganic phosphorus-dissolving, and iron-producing carriers. (3) Results: The dominant bacterial phyla were found to be Proteobacteria and Acidobacteria, and *unclassified_Rhizobiales* was the most abundant genus. Ascomycota and Mortierellomycota were the dominant fungal phyla. The bacteria were mainly carbon and nitrogen metabolizers, and the fungi were predominantly Saprotroph—Symbiotroph. The identified network was completely dominated by positive correlations, but the fungi were more complex than the bacteria, and the bacterial keystones were *unclassified_Caulobacteraceae* and *Pedobacter*. Most of the keystones of fungi belonged to the phyla Ascomycetes and Basidiomycota. The highest number of different species of culturable bacteria belonged to the genus *Streptomyces*, with three strains producing IAA, 12 strains solubilizing organic phosphorus, one strain solubilizing inorganic phosphorus, and nine strains producing iron carriers. (4) Conclusions: At the cost of reduced ecological stability, microbial communities increase cooperation toward promoting overall metabolic efficiency and enabling their survival in the extreme environment of the Tibetan Plateau. These pioneering results have value for the protection of endangered *Meconopsis integrifolia* under global warming and the sustainable utilization of its medicinal value.

## 1. Introduction

Chinese medicine has developed in China over thousands of years, and it currently plays a unique role in the prevention and control of novel coronavirus pneumonia [1], so it is necessary and worthwhile to pay attention to Chinese medicinal plants. *Meconopsis integrifolia* is an annual or perennial herb of the genus *Meconopsis* in the poppy family, a snowy mountain beauty from the extreme environment of the Tibetan Plateau [1,2] which has high ornamental value due to its colorful flowers [3] and is widely used in gardening and as a customary Tibetan medicinal herb [4]. Alkaloids and flavonoids can be isolated from species of the genus *Meconopsis*, which have anti-inflammatory and analgesic properties [5,6,7,8,9,10,11,12]. *Meconopsis integrifolia* has been used as a classical Tibetan medicine, “Gopala”, which is used for the treatment of pneumonia, hepatitis, headache, and edema, among others [4]. However, in recent years, due to global climate change, habitat destruction, human activities [13,14], and overexploitation of wild resources, the survival and sustainability of *Meconopsis integrifolia* have been seriously threatened, and the country has listed *Meconopsis integrifolia* as a class I endangered Tibetan medicine [15]. Due to the strict requirement of its particular environment (3400–5000 m above sea level) and the difficulty of seed germination [16,17,18,19], artificial cultivation is faced with the challenge of adapting to various conditions, such as temperature and soil, that are different from its native habitat [20,21], all of which represent barriers for its future horticultural and medicinal use. Although artificial cultivation of some species of the genus *Meconopsis* has been shown to be successful in other countries [22], and attempts to artificially cultivate *Meconopsis* plants have been reported in individual research units such as the Beijing Botanical Garden [23] and the Tibetan Hospital of the Tibet Autonomous Region [24] in China, a limited number of species were investigated, and artificial cultivation of *Meconopsis* is still not widely implemented because of many unanswered questions.

In a previous study, Yu et al. [25] have basically optimized the main technical parameters such as temperature, humidity, and soil substrate required for the growth and development of *Meconopsis integrifolia* from seed stage to seedling stage at 3000 m altitude. However, *Meconopsis integrifolia* is characterized by a stable root microbial community and slow root development, which makes it difficult for it to recover after damage, and it shows poor adaptation to substrate changes and following transplantation, with relatively few viable plants produced in the field. As a result, direct transplanting of seedlings has great risk and represents a technical bottleneck. In China, some progress has been made in *Meconopsis* tissue culture. For example, it is feasible to propagate *Meconopsis horridula* in large numbers through the use of tissue cultures following sterile seeding [26]. These findings suggest that the growth of *Meconopsis* may be influenced by some specific factors, especially soil microorganisms, and this aspect is currently unknown.

In recent years, it has been found that soil microbial ecology affects the health of plants and has a catalytic effect on plant growth through either promoting plant growth via the aggregation of beneficial microorganisms [27] or inhibiting pathogenic bacteria to improve the adaptation of plants to their environment [28]. Specific microorganisms can assemble into plant-associated communities that affect the growth and health of the host plant [29]. The Tibetan Plateau (known as “the third pole”) is characterized by severe cold and anoxia, large diurnal temperature differences, high radiation, and long daylength [30]. High-altitude soils are unique and may pose a challenge to life, mainly because of their oligotrophic properties [31] and intense freeze–thaw cycles, even in summer [32]. Extreme environments have prompted plant species to evolve and strongly adapt, thus shaping microbial communities. Previously, Fan et al. studied the endophytic and rhizospheric actinomycete diversity of the medicinal plant *Meconopsis punicea Maxim*. using culturable and culture-free PCR-DGGE techniques [33]. However, studies on soil microorganisms in artificial cultivation of *Meconopsis integrifolia* have not been reported, especially details of the microbial interactions in soils, which remain unknown.

High-throughput sequencing technology has the characteristics of faster, accurate, scientific, and comprehensive probing of sample microbial community structure [34]. In this experiment, we used the second-generation high-throughput sequencing technology based on Illumina MiSeq platform to sequence the 16S rRNA and ITS genes of soil microbial of *Meconopsis integrifolia* for the first time to reveal the diversity and structure of the soil microbial community and predict function, in addition to elaborating the microflora inter-relationships. This was conducted in combination with culturable techniques to isolate and identify functional bacteria, which provides a scientific basis for further investigation and screening of the core functional microbial mixes in flora that are involved in maintaining the growth of *Meconopsis integrifolia* and improving the wild-like soil of cultivated *Meconopsis integrifolia* in regulating the microbial environment and helping to promote artificial cultivation of *Meconopsis integrifolia*. 

## 2. Materials and Methods

### 2.1. Study Area and Sampling

In this study, potting soil of artificial cultivated *Meconopsis integrifolia* was collected. The experimental site was located at the cultivation base of rare and endangered plants of Tibet Agricultural and Animal Husbandry University, Nyingtri City, Tibet Autonomous Region, 29°67′ E, 94°38′ N, at an altitude of 3000 m. The site has a highland temperate humid–semi-humid monsoon climate, with annual precipitation of about 650 mm, average annual temperature of 8.7 °C, and average annual sunshine hours of about 1988 h, with a sunshine percentage of 46% and a frost-free period of about 180 days. Yu et al. sowed the seeds of *Meconopsis integrifolia* in mid-to-late November 2019 near the native colony at 4500 m elevation in the Shergyla Mountain to break dormancy by cold stratification. The white seeds were retrieved in mid-June 2020 after they had partially emerged. In an artificial climate box, the exposed white seeds were placed in a petri dish with two layers of moist sterile filter paper and evenly spread. The seeds were then cultivated under the conditions of moist filter paper and enough light, and the young buds were then sown in pots until the seeds germinated. The seedling cultivation experiment was carried out under the condition of sufficient light and moderate soil moisture content.

*Meconopsis integrifolia* planted in 2 pots (20 cm × 20 cm × 20 cm) survived and demonstrated robust growth, and soil samples were collected from each of the pots in November 2020. For sampling, 3 points around 2 cm from the roots and 3 points 2 cm from the pot edge were collected at a depth of 10 cm by hand with use of sterile gloves. Soil from these 6 points was mixed, and a final total of 2 samples were packed in two separate sterilized bags for each of the pots. Freshly collected samples were placed on dry ice for immediate transport to the laboratory. In the laboratory, roots, plant residues, stones, and any other debris were removed from the soil through a 2 mm sieve. Each soil sample was divided into 4 equal portions to obtain a total of 8 soil samples. Samples from one pot were numbered M1, M2, M3, and M4, and from the other were numbered M5, M6, M7, and M8. The soil was stored at −80 °C and then subjected to DNA extraction before data analysis.

### 2.2. DNA Extraction, PCR, MiSeq Sequencing, and Sequence Data Analysis

Total genomic DNA was extracted using the E.Z.N.A^TM^ Mag-Bind soil DNA kit (OMEGA, Norcross, GA, USA) according to the manufacturer’s instructions. DNA integrity was examined by 1% agarose gel electrophoresis, and DNA concentration was quantitatively detected using Qubit. The V3–V4 region of the bacterial 16S rRNA genes was amplified using primers 341F (5′-CCTACGGGNGGCWGCAG-3′) and reverse primer 805R (5′-GACTACHVGGGTATCTAATCC-3′), and the ITS1 region of the transcribed spacer was amplified using primers ITS1F (5′-CTTGGTCATTTA GAGGAAGTAA-3′) and ITS2R (5′-GCTGCGTTCTTCATCGATGC-3′) for fungal community analysis. 16S rRNA genes were amplified using specific primers with barcodes. The first round PCR reactions were conducted in volume of 30 μL containing 15 μL of 2 × Hieff Robust PCR Master Mix, 1 μL each of Bar-PCR primer F and primer R, and 10–20 ng of template DNA. The PCR amplification cycle consisted of an initial denaturation at 94 °C for 3 min. This was followed by five cycles of 94 °C denaturation for 30 s, annealing at 45 °C for 20 s, and extension at 65 °C for 30 s, followed by 20 cycles of denaturation at 94 °C for 20 s, annealing at 55 °C for 20 s, and extension at 72 °C for 30 s, and final extension at 72 °C for 5 min. In the second round of amplification, Illumina bridge PCR-compatible primers were introduced. The reaction system was the same as the first round with the exception that the primers were replaced by Primer F and Index-PCR Primer r. Library quality was assessed using a Qubit@3.0 fluorometer (Thermo Scientific, Waltham, MA, USA). Finally, the libraries were sequenced on the Illumina MiSeq platform to generate 250 bp paired-end reads. 

### 2.3. Sequence Processing and Statistical Analysis

Sequences in the library were clustered using USearch, operational taxonomic units (OTUs) were clustered at 97% similarity for non-repetitive sequences (excluding individual sequences), and chimeras were removed during clustering to obtain representative sequences of OTUs [35,36,37]. Then, each representative sequence was labeled with taxonomic information using the RDP classifier. In calculating alpha diversity, we calculated two indices, ACE index and Shannon evenness index, to estimate the species richness and species evenness of the communities, respectively. The 10 most abundant phyla were selected from all samples included in the phylum-level species annotation and abundance information. The columnar stack diagram of relative abundance of each sample was plotted using R software. The 40 genera with the highest abundance were selected for the heatmap. To further explore the microbial community, we used the PICRUSt [38], FAPROTAX [39], and FUNGuild [40] annotation tools for functional prediction. KEGG microbial metabolic functions and FUNguild were used to demonstrate abundance greater than 50 via https://www.chiplot.online/ (accessed on 4 September 2022). The co-occurrence network was analyzed with R and visualized using the Gephi interaction platform and the Fruchterman–Reingold layout algorithm [41].

### 2.4. Isolation and Identification of Functional Bacteria

#### 2.4.1. Medium and Reagents

Beef extract peptone agar (NA) and R2A medium were used for bacterial isolation, purification, and preservation. Manginella organophosphorus medium was used for the identification of the solubilization capacity of organic phosphorus [42]. PKO (Pikovaskaias) inorganic phosphorus medium [43] was used for the identification of the solubilization capacity of inorganic phosphorus. Chrome azurol S (CAS) assay medium was used for the identification of the capacity of iron carriers [44]. The colorimetric solution for the IAA colorimetric reaction was formulated as described in [45].

#### 2.4.2. Experimental Methods

##### Isolation and Identification of Bacteria from Soil Samples

Eight soil samples were mixed, and 10 g of soil was accurately weighed and placed in a conical flask with 100 mL sterile water with pre-sterilized glass balls bearings. The conical flask was shaken at 150 r/min for 30 min at 30 °C to obtain a soil suspension, which was then allowed to stand for 30 min. Dilutions of 10^−1^, 10^−2^, 10^−3^, 10^−4^, and 10^−5^, representing a concentration gradient, were prepared by taking 1 mL supernatant and added to 9 mL sterile water test in series. The dilutions of 10^−3^, 10^−4^, and 10^−5^ were selected, and 100 μL of dilutions were pipetted and mixed on beef extract peptone agar and R2A medium, respectively. Three parallels of each dilution were cultivated upside down in an incubator at 30 °C. From day five, colonies were selected according to their phenotypic characteristics (including size, shape, color, texture, etc.) [46] and purified by dilution streaking on medium. After purification, the strains were subjected to 16S rRNA gene amplification. The PCR amplification products were sent to Beijing Biomed Gene Technology Company for sequencing. Sequencing results were submitted to http://ezbiocloud.net (accessed on 4 September 2022) for comparison. Finally, the bacteria were preserved in a −80 °C refrigerator with 60% glycerol. 

##### Functional Identification

The purified bacteria were made into bacterial suspensions and cultured overnight. Suspensions (10 µL) were inoculated in organophosphorus, inorganic phosphorus, and CAS media. Three replicates of each bacteria were inoculated and cultivated at 30 °C. Organophosphorus and inorganic phosphorus media were visually assessed for the appearance of transparent circles, and CAS media for the appearance of yellow-green haloes. On day seven, the capacity of each strain to dissolve organophosphorus and inorganic phosphorus and produce iron carriers was determined based on the diameter of the formed circles as the circle diameter (D)/colony diameter (d) [47].

Determination of IAA production capacity [45,48]: Isolated and purified bacteria were inoculated in 50 mL of NA liquid medium containing L-tryptophan (100 mg/L) and incubated for 1 d at 30 °C, 180 r/min. (1) Qualitative determination: 50 μL of bacterial suspension was added dropwise to a white ceramic plate with an equal volume of Salkowski colorimetric solution (50 mL 35% HClO_4_ + 1 mL 0.5 mol/L FeCl_3_). The white ceramic plate was left at room temperature and protected from light for 30 min. Red color indicated the ability to secrete indoleacetic acid. (2) Quantitative determination: 10 mL of bacterial suspension were centrifuged at 10,000 r/min for 10 min to obtain 2.5 mL of supernatant, and IAA standard solutions were prepared with concentrations of 0, 10, 20, 30, 40, 50, and 60 μg/mL. The bacterial suspension was mixed with Salkowski colorimetric solution at a volume ratio of 1:1. The OD530 values were measured after 30 min of incubation under low light. The IAA concentrations of N1-12, N1-21, and N1-73 were obtained according to the standard curve.

## 3. Results

### 3.1. Soil Microbial Diversity 

A total of 781,611 16S rRNA gene valid sequences and 409,670 ITS valid sequences were identified from the raw sequence reads. In addition, 3848 bacterial OTUs and 1197 fungal OTUs were aggregated from these valid sequences. The complexity of the sample species diversity was analyzed using α-diversity indices, including richness indices (ACE and Chao1) and community diversity indices (Shannon and Simpson) as indicators. The alpha diversity indices of bacterial and fungal communities are shown in Table 1. The diversity of bacteria was greater than that of fungi.

### 3.2. Soil Bacterial and Fungal Structure 

**Figure 1:**
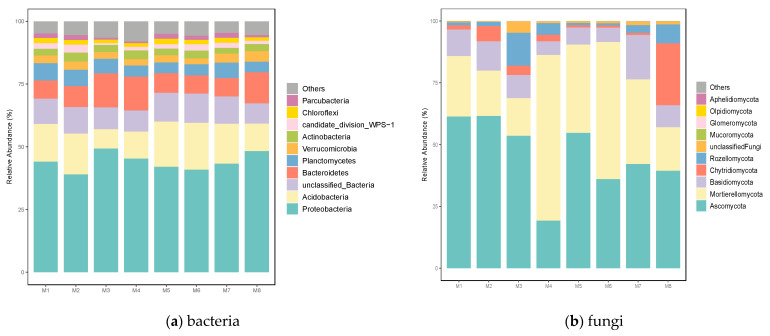
Differences in the relative abundance of (**a**) bacterial and (**b**) fungal phyla. “Others” includes phyla with less than 0.1% of relative abundance.

The predominant phyla of bacterial communities were found to be Proteobacteria (39.03%–49.31%), Acidobacteria (7.74%–18.67%), unclassified_Bacteria (8.09%–11.67%), Bacteroidetes (7.25%–13.55%), Planctomycetes (4.22%–6.89%), Verrucomicrobia (2.28%–4.20%), Actinobacteria (2.32%–3.53%), candidate_division_WPS−1 (0.75%–3.17%), Chloroflexi (1.35%–2.16%), and Parcubacteria (0.77%–1.98%), representing more than 50% of the total sequences (Figure 1). Ten predominant phyla (with relative abundance higher than 0.01%) of fungal communities were identified: Ascomycota (19.33%–61.41%), Mortierellomycota (15.31%–66.87%), Basidiomycota (5.51%–18.00%), Chytridiomycota (0.86%–25.06%), Rozellomycota (0.91%–13.35%), unclassified_Fungi (0.21%–4.59%), Mucoromycota (0.02%–0.08%), Glomeromycota (0.008%–0.05%), Olpidiomycota (0.00%–0.04%), and Aphelidiomycota (0.00%–0.04%), representing more than % of the total sequences (Figure 1). Ascomycota and Mortierellomycota accounted for the majority, representing about 75%.

**Figure 2:**
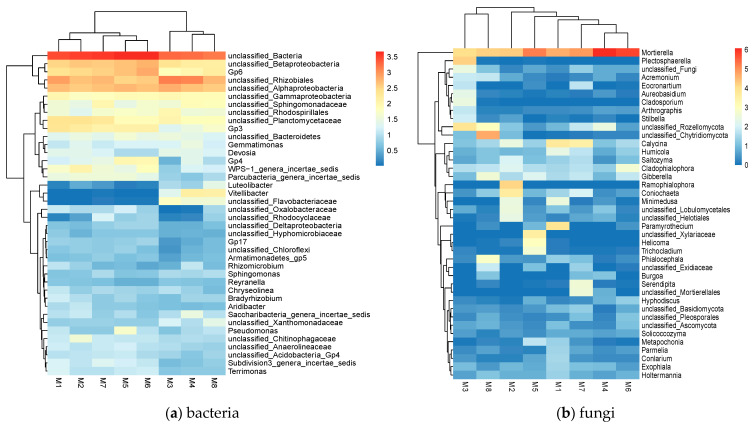
Heatmaps of top 40 genera of soil (**a**) bacterial and (**b**) fungal communities. Relative abundance of soil microbial community from high to low is represented by red, yellow, and blue.

The 40 most abundant genera of soil bacterial and fungal communities can be seen in Figure 2. The relative abundance of soil microbial community from high to low is represented by red, yellow, and blue. For bacterial communities, the predominant genera are *unclassified_Rhizobiales*, *unclassified_Alphaproteobacteria*, *unclassified_Betaproteobacteria*, *Gp6*, *unclassified_Planctomycetaceae*, *Gp3*, *unclassified_Gammaproteobacteria*, *unclassified_Sphingomonadaceae*, *unclassified_Rhodospirillales*, *unclassified_Bacteroidetes*, *Gemmatimonas*, and *Devosia*. The predominant genera of fungal communities are *Mortierella*, *Calycina*, *unclassified_Rozellomycota*, *unclassified_Chytridiomycota*, *Cladophialophora*, *Coniochaeta*, *Gibberella*, *Plectosphaerella*, *Ramophialophora*, *Paramyrothecium*. Soil bacteria and fungi with annotated information in *Meconopsis integrifolia* only account for a small fraction of the sequencing data, which suggests that the currently available prokaryotic reference genomes do not meet the requirements for annotation of microbial species in samples of *Meconopsis integrifolia* soil.

### 3.3. Function of Soil Bacterial and Fungal Community

#### 3.3.1. Prediction of Bacterial Community Function

**Figure 3:**
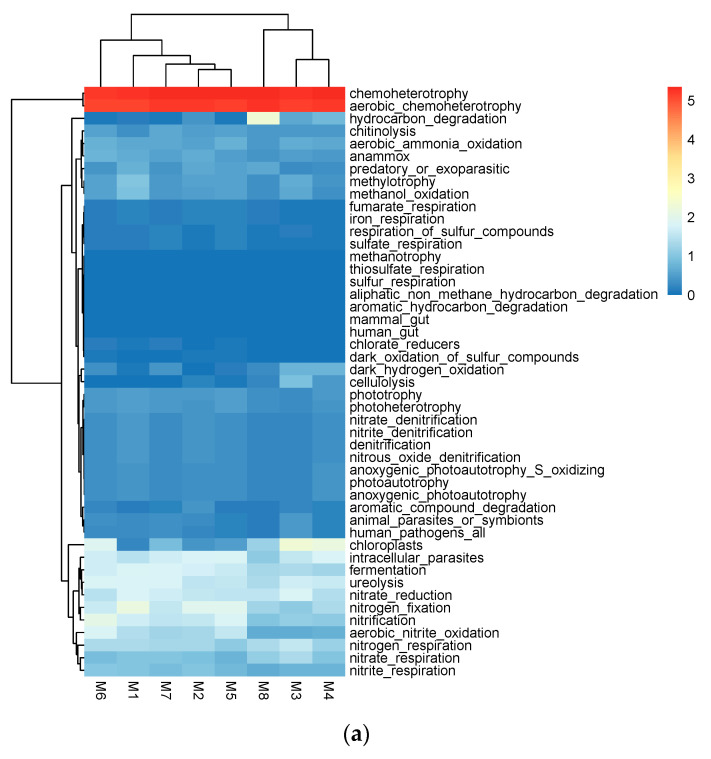

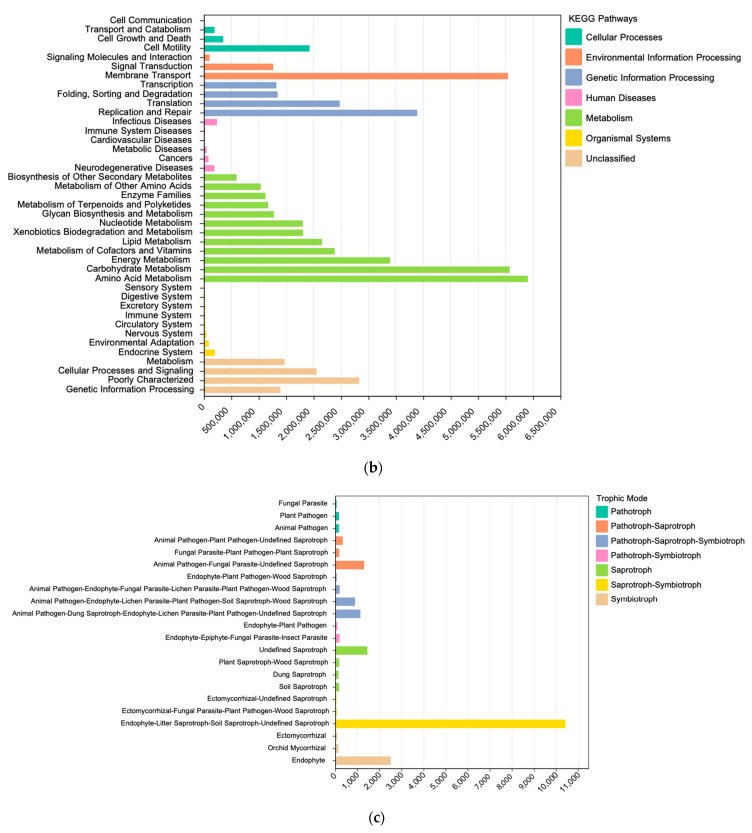
(**a**) Heatmap of 47 functions after functional annotation of the FAPROTAX community. (**b**) Column classification of KEGG metabolic pathways, showing the abundance values of metabolic pathways for 8 samples. (**c**) Classification of fungal trophic patterns after annotation by FUNguild function.

A total of 47 functional population groupings were obtained after functional annotation of the soil bacterial community using FAPROTAX (Figure 3a). Among them, 10 functional types with high relative abundance and their total relative abundance accounted for more than 22% of the total abundance, and the functions attributed to others (54%–74%) are still unclear (Figure 3b). Chemoheterotrophy was the most abundant identified function, accounting for more than 8% of the total abundance, followed by ureolysis, nitrogen_fixation, intracellular_parasites, nitrate_reduction, and fermentation. Reduction, fermentation, chloroplasts, nitrification, and nitrogen_respiration were detected at an abundance above 0.1%. Functional populations of mammalian intestinal gut and human gut were also detected.

The metabolic functions of the community samples were predicted by PICRUSt software based on the categories of microbial metabolic functions in the KEGG database. By comparing the KEGG database, six types of biometabolic pathways were obtained, including metabolism, genetic information processing, environmental information processing, and cellular processes. The metabolic pathways are divided into 37 subfunctions (Figure 3c). At the primary functional level, the metabolic system accounts for the largest share (50.93%). At the secondary functional level, amino acid metabolism, carbohydrate metabolism, and energy metabolism account for 10.66%, 10.06%, and 6.12%, respectively. Special attention is paid to the metabolism of terpenoids and polyketides at 2.11%. The largest proportion of genetic heritage information processing is 10.00% for replication and repair. Membrane transport accounts for 7.02% of environmental information processing. The organism system contains 0.36% of endocrine system, 0.16% of environmental adaptation, 0.09% of nervous system, and 0.05% of circulatory system. Among the cellular processes, the mobility of cells was, at its maximum point, 3.84%. Among human diseases, infectious diseases account for the largest at 0.43%, followed by neurodegenerative diseases at 0.35%, cancer at 0.15%, metabolic diseases at 0.09%, and immune system diseases at 0.04%.

#### 3.3.2. Prediction of Fungal Community Function

**Figure 4:**
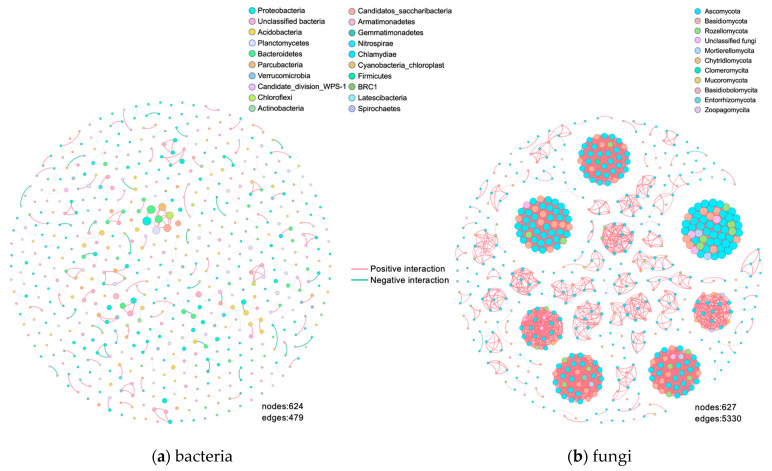
(**a**) Bacterial and (**b**) fungal network of co-occurring OTUs based on correlation analysis. The nodes in the figure represent OTUs, and the edges (lines between two nodes) indicate the existence of correlation between two OTUs (spearman *p* > 0.6, *p* < 0.01). The more lines, the closer the connection between the species and other species. The node size indicates the size of species connectivity degree. The color indicates the same phylum. The thickness of the edge represents the correlation size. The color of lines represents the positive and negative correlation: pink represents positive correlation, green represents negative correlation. Only OTUs with at least 50% recurrence in each sample are shown in the figure.

Functional prediction of fungal communities using FUNGulid (Figure 4) shows that 558 out of 1197 OTUs were classified into seven fungal functional taxa, accounting for about 46.62% of the total OTUs, namely Symbiotroph, Saprotroph, Pathotroph, Saprotroph–Symbiotroph, Pathotroph–Symbiotroph, Pathotroph—–Saprotroph, Pathotroph–Saprotroph–Symbiotroph. As shown in the figure, the largest proportion was Saprotroph––Symbiotroph 51.66%, where Endophyte–Litter, Saprotroph–Soil, and Saprotroph–Undefined Saprotroph were dominant.

### 3.4. Analysis of Intra-Domain Co-Occurrence Networks

In the bacterial co-occurrence network (Figure 4a), the pink edges indicate positive correlations with 77.24%, and the green edges indicate negative correlations with 22.76%, indicating that interaction between bacteria in this study was largely positive. Proteobacteria, Unclassified Bacteria, Acidobacteria, Planctomycetes, and Bacteroides were dominant in all bacterial networks. There were 237 modules in the network, of which two green nodes OTU1520 (*unclassified_Caulobacteraceae*, degree = 8) and OTU2821 (*Pedobacter*, degree = 8) were the most connected and defined as core nodes in the network. The edge is pink with a higher weight, and a strong positive correlation is observed between the two. OTU786 (*unclassified_Flammeovirgaceae*, degree = 7), OTU2678 (*unclassified_Sphaerobacteraceae*, degree = 7), OTU1352 (*unclassified _Planctomycetaceae*, degree = 7), OTU16 (*unclassified_Cyanobacteria*, degree = 7), and OTU1045 (*Saccharibacteria_genera_incertae_sedis*, degree = 7) have the next highest degree of connectivity. The edges are pink and coarse, indicating a relatively strong positive correlation. OTU450 (*unclassified_Bacteria*, degree = 5), OTU88 (*unclassified_Myxococcales*, degree = 5), and OTU807 (*unclassified_ Chitinophagaceae*, degree = 5) were highly connected with OTU107 (*Armatimonadetes_gp5*, degree = 5), where they exhibit a strong positive correlation. OTU2910 (*Altererythrobacter*, degree = 4) and OTU434 (Gp17, degree = 4) exhibit a strong negative correlation. OTU899 (*unclassified_Chitinophagaceae*, degree = 4) and OTU150 (*Pedobacter*, degree = 4) also show a relatively strong negative correlation.

In the fungal co-occurrence network (Figure 4b), positive correlations account for 99.89% and negative correlations account for 0.11%, which indicates higher complexity (based on the increased number of edges and nodes) compared with bacteria. Most of the nodes in the fungal network are mainly from Ascomycota, Basidimycota, Rozellomycata, and unclassified fungi. There are 121 modules in the fungal network, of which there are seven larger modules, and all OTUs in the modules are positively correlated with each other. The OTUs in the modules with the highest connectivity of 55 are all defined as core nodes and come from four phyla. Among them, Ascomycota includes OTU13 (*unclassified_xylariaceae*), OTU 110 (unclassified_Didymellaceae), OTU154 (*Hamatocanthoscypha*), OTU212 (*unclassified_ Sympoventuriaceae*), OTU 238 (*unclassified_Rutstroemiaceae*), OTU468, OTU700, and OTU1027 (*unclassified_Helotiales*), OTU515 (*Alatospora*), OTU578 (*Eleutheromyces*), OTU667 (*Fusidium*), OTU748 (*Preussia*), OTU819 (*unclassified_Herpotrichiellaceae*), OTU873 (*Phaeosphaeria*), OTU947 (*Curvularia*), OTU946 (*Archaeorhizomyces*), OTU1018 (*Ascobolus*), OTU949 (*Sporormia*), OTU1020 (*Candida*), OTU1023 OTU1259 (*Archaeorhizomyces*), OTU1112 (*Coniochaeta*), OTU1114 (*Dothiora*), OTU1115 (*unclassified_Capnodiales*), OTU1256 (*Conlarium*), OTU1265 (*Cephaliophora*), OTU1266 (*unclassified_Microthyriales*), OTU1505 (*Myrothecium*), OTU1506 (*unclassified_Chaetothyriales*), OTU1507 (*Hymenoscyphus*), OTU1478 and OTU1870 (*Archaeorhizomyces*), OTU1484 (*Collophora*), OTU1485 (*Tothia*), OTU1488 (*Duddingtonia*), OTU1876 (*Cryptodiscus*), OTU1892 (*Simplicillium*), and OTU1899 (*Beauveria*).The phylum Mortierellomycota includes OTU403 and OTU1260 (*Mortierella*). The phylum Basidiomycota includes OTU195 (*Waitea*), OTU516 (*Calocera*), OTU637 (*Coprinus*), OTU874 (*Trichosporon*), OTU945 (*Sakaguchia*), OTU1257 (*Serendipita*), and OTU1270 (*Tomentella*). The Rozellomycota phylum includes OTU1264 and OTU1113 (*unclassified_GS11*), OTU1116, OTU1482, and OTU1886 (*unclassified_Rozellomycota*), and OTU1508, OTU1487, and OTU1261 (*unclassified_Fungi*). Only six edges exhibit negative correlation, including OTU42 (*Stilbella*) and OTU 239 (*unclassified_Myrmecridiales*); OTU62 (*unclassified_Basidiomycota*) and OTU 809 (*Bradymyces*); OTU 437 (*Fusarium*) and OTU 488 (*Russula*); OTU 298 (*Podospora*) and OTU 422 (*Mortierella*); OTU 308 (*Cladophialophora*) and OTU 418 (*Cystobasidium*); and OTU 542 (*Paraphaeosphaeria*) and OTU 2042 (*Cladophialophora*). The nodes with the highest degree of connectivity were defined keystones and are also key microorganisms in the soil of *Meconopsis integrifolia*.

Table 2 summarizes the main topological features of the co-occurrence network. The number of nodes and edges is higher in the fungal network than the bacterial network. The density of the fungal network is 0.027, which is much higher than that of the bacterial network’s 0.002. The fungal co-occurrence network is more complex and stable than that of the bacterial network. The average connectivity of 17.002 for fungi is higher than bacteria’s 1.535, and the average clustering coefficient of 0.967 for fungi is higher than bacteria’s 0.622.

### 3.5. Culturable Bacteria

A total of 49 species of bacteria belonging to 18 genera were isolated from the soil (Table S1). Among these bacteria, Actinobacteria had the highest abundance, accounting for 34.69% of the total strains, followed by Proteobacteria (26.53%), Firmicutes (20.40%), and Bacteroidetes (18.37%). *Streptomyces* spp. in the phylum Actinobacteria accounts for 70.59%.

Strains marked with N indicate screening from beef extract peptone agar and strains marked with R indicate screening from R2A medium. Three of the isolated strains had the ability to produce IAA. They are N1-12 (*Pseudomonas laurylsulfatiphila*), N1-21 (*Microbacterium algeriense*), and N1-73 (*Peribacillus simplex*) (Table 3). The IAA standard curve is shown in Table S2. Among the strains, N1-73 has the strongest ability to produce IAA. There were twelve organophosphate-solubilizing strains found (Table 4), among which R2-10 (Streptomyces murinus), N1-72 (*Microbacterium phyllosphaerae*), and N1-73 (*Peribacillus simplex*) have the strongest organophosphate-solubilizing ability. In Table 4, D denotes the diameter of the transparent circle and d denotes the diameter of the bacteria. Nine strains produce iron carriers, with N1-72 (*Microbacterium phyllosphaerae*) and N1-6 (*Streptomyces thermoviolaceus* subsp. *Thermoviolaceus*) having the strongest ability in this regard. The one strain that solubilizes inorganic phosphorus is N1-12 (*Pseudomonas laurylsulfatiphila*). N1-12 has the ability to produce IAA, soluble organic phosphorus, soluble inorganic phosphorus, and iron carrier at the same time. N1-21 has the ability to produce IAA and iron carrier. N1-73 has the ability to produce IAA and soluble organic phosphorus. R2-10 (*Streptomyces murinus*), R2-18 (*Streptomyces rishiriensis*), N1-6 (*Streptomyces thermoviolaceus subsp. Thermoviolaceus*), and N1-72 (*Microbacterium phyllosphaerae*) have both organophosphorus- and iron-carrier-producing abilities. Specific information on each strain is presented in Table S3.

## 4. Discussion

### 4.1. Community Structure of Soil Microorganisms

#### 4.1.1. Structural and Functional Prediction of Bacteria

In the soil microbial community of artificial cultivated Meconopsis integrifolia, the dominant bacterial groups were mainly distributed in Proteobacteria, Acidobacteria, Bacteroidetes, Planctomycetes, Verrucomicrobia, Actinobacteria, and Chloroflexi. Proteobacteria were the dominant species in different geographical areas and soil types, which is consistent with the conclusion that species of the phylum Proteobacteria are the most abundant in this study. Proteobacteria play an important role in nitrogen utilization, control of plant diseases, and soil remediation [49]. Guan et al. [50] found that the dominant bacterial groups in alpine meadow ecosystems of the Tibetan Plateau are Proteobacteria, Actinobacteria, Bacteroidetes, Acidobacteria, and Verrucomicrobia, which is consistent with the results of this study. Alonso et al. [51] found that Acidobacteria and Chloroflexi, which have a high probability of occurrence and account for a large proportion of the population in the Qinghai–Tibetan Plateau, hardly ever occur in the Antarctic continent. Acidobacteria and some Chloroflexi have a large number of functional genes related to carbon degradation and organic repair [52], thus playing an important role in organic matter decomposition and nutrient cycling processes. Interestingly, it has been found that Acidobacteria and some Chloroflexi are substantially reduced after warming, which may reduce their overall carbon utilization capacity [53]. This may be one of the reasons why the Meconopsis from high altitudes cannot colonize low altitudes in the Tibetan Plateau. Verrucomicrobia, Chloroflexi, and Planctomycetes are synergistically involved in cellulose decomposition [54]. Bradyrhizobium in the dominant genus of bacterial communities, and use of Rhizobiales (top1) as a solid nitrogen inoculant or bioorganic fertilizer has been reported [55]. It can be used to produce bacterial fertilizer for astragalus [56]. GP3 and GP6 belong to Acidobacteria. They have the highest abundance in soils with very low resource availability [57], which may explain how Meconopsis integrifolia adapts to harsh oligotrophic soil environments, namely by recruiting more Acidobacteria to break down organic matter and promote nutrient cycling. Alphaproteobacteria contain a large number of other nitrogen-fixing bacteria that are symbiotic with plants [58]. Sphingomonas are organic compound-degrading bacteria that are soil probiotics that can improve plant stress tolerance [59]. Gemmatimonadota can survive in extreme environments. The ability of some members to access light can increase their growth rate [60] while increasing the rate of assimilation of organic compounds [61]. Certain strains of Devosia have potential for mycotoxin degradation and are known for their bioremediation potential [62]. 

Across all bacterial samples, chemoheterophy was found to be a predominant function, followed by aerobic chemoheterotropy, both of which are considered widespread ecosystem functions performed by most microorganisms [63], including Acidobacteria, Proteobacteria, and Verrucomicrobia [64]. These are the dominant bacterial phyla in *Meconopsis integrifolia* soil. Moreover, chemoenergetic, aerobic chemoheterotropy, and fermentation are important ecological functions associated with carbon cycling [65]. This indicates that a higher percentage of bacteria in *Meconopsis integrifolia* soils are involved in the soil carbon cycle, which facilitates the release of many host-available inorganic nutrients from organic matter. Other functions related to the nitrogen cycle such as ureolysis, nitrogen fixation, nitrate reduction, nitrification, and nitrogen respiration indicate active nitrogen metabolism in soil. Surprisingly, the functional taxa *Rhizobiales*, associated with the nitrogen cycle, is the most abundant genus of soil bacteria in *Meconopsis integrifolia*, including *Devosia*, *Bradyrhizobium*, *Microvirga*, *Mesorhizobium*, and *Phyllobacterium*. Given the limitations of PICRUSt functional prediction, the large proportion of many unexplored functions indicates that there is still a great potential for exploration. Metabolism is the most important function in the growth of *Meconopsis integrifolia*, where amino acid metabolism is closely related to the plant nitrogen cycle [66]. It has also been shown that carbohydrate metabolism is closely related to nitrogen and phosphorus in the plant, and an increase in nitrogen and phosphorus cycle can promote plant carbohydrate metabolism [67]. All of the above indicate that the soil bacteria of *Meconopsis integrifolia* mainly metabolize carbon and nitrogen, degrade organic matter, promote nutrient cycling, and create an environment that is favorable for host survival. It is also worth noting that the presence of metabolism of terpenoids and polyketides indicates that some *Meconopsis integrifolia* soil bacteria contain natural active substances, thus unveiling a window of opportunity for the medicinal use of *Meconopsis integrifolia* soil microorganisms.

#### 4.1.2. Structural and Functional Prediction of Fungi 

Ascomycota and Mortierellomycota were found to be the first and second most dominant fungal groups, respectively. Zhang et al. found that Ascomycota, Zygomycota, and Basidiomycota are dominant in meadows and grasslands of the Tibetan Plateau [68], which is consistent with the findings here. One study reported that unlike other soil systems, high altitude soils are dominated by *Chytridiomycota chytrids*, and these fungi, with an aquatic dispersal (flagellar) phase of reproduction, are dependent on soil moisture from melting snowpacks, especially for the endogenous primary production of microbial phototrophs [69]. Among them, members of Spizellomycetales and its sister order Rhizophlyctidales [70,71] both have significant freezing and drying tolerances [72], and members of Spizellomycetales are usually saprophytic. This is consistent with the climatic and environmental conditions for the growth of artificial cultivated *Meconopsis integrifolia*, indicating the uniqueness of the environment required for its growth. Very noteworthy is Glomeromycota, as genera of this phylum are classified as AMF. It is well known that AMF plays a role in the health of the host plant through several means, including by activating the defense mechanisms against soil-borne diseases and effectively absorbing phosphorus in soil and transferring it to plants [73], and AMF are characterized by rapid colonization of habitats without competitors. The fungal community dominant genus *Mortierella* spp. (top1) is saprophytic and cold tolerant fungi. They are the most abundant filamentous fungi in soils worldwide and very promising plant growth promoters in agriculture [74,75]. Strains of *Clavaria* may also play a role as deep humic decayers [76]. The fruiting bodies of *Clavaria* are also of interest for their content in antioxidant compounds and essential trace elements, which are beneficial to human health [77]. Up to now, chemical components in secondary metabolites from *Cladosporium* have been isolated and identified, including alkaloids, polyketides, macrolides, steroids, and terpenes. Most possess antimicrobial, antivirus, and cytotoxic activities [78]. *Gibberella* can produce gibberellin, which has beneficial effects on agricultural crops [79]. Some patents reported the strain *Ramophialophora* has a good growth-promoting effect on sugarcane and tomatoes, reduces the severity of rice disease, and improves the resistance of rice to dwarf disease [80]. Some strains of *Humicola* have shown potential as bio-organic fertilizers or as biocontrol organisms for plant diseases [81,82]. Notably, being annotated as unclassified shows that many of the soil microorganisms in the extreme environment of the Tibetan Plateau are unknown, but also implies a great potential for exploitation. The largest fungal proportion of Saprotroph–Symbiotroph indicates that fungi in *Meconopsis integrifolia* soils are mainly focused on decomposing organic matter, especially via mycorrhizal fungi that establish symbiosis with plant roots, which greatly enhances the ability of *Meconopsis integrifolia* to survive in harsh environments. 

### 4.2. Co-Occurrence Network

In the natural environment, microorganisms form microbial networks through a series of direct and indirect ecological interactions such as synergy, competition, and antagonism [83,84], representing the basis by which ecosystems achieve the basic functions of energy flow, material cycling, and information transfer [85]. Microbial co-occurrence networks can reveal complex interactions among microorganisms and can reflect ecological connections and ecological processes not reflected by species diversity [86,87].

#### 4.2.1. Fungal Networks Are More Complex and More Sensitive to External Disturbances Than Bacterial Networks

The mean degree, network density, and mean clustering coefficient of soil fungi were found to be greater than those of bacteria, indicating that fungal communities in *Meconopsis integrifolia* soils are more complex than bacteria. Zhang et al. took the alpine meadow on the Tibetan Plateau as the research object and found that, compared with microbial species, network complexity can better predict ecosystem versatility. Microbial diversity indirectly and positively influences ecosystem versatility by promoting network complexity [88]. It is hypothesized that the network of soil fungi of *Meconopsis integrifolia* can enhance ecosystem multifunctionality. It has been shown that complex soil microbial community networks (networks with a high number of nodes, connections, and average connectivity) are beneficial to plants [89]. For example, tobacco rhizospheric microbial communities with complex networks have a very low incidence of bacterial wilt [90]. Fungi in the *Meconopsis integrifolia* soil may play an important role in the resistance to diseases. Microbial communities with more complex networks have more efficient resource use cycles and information transfer processes than those with simpler networks [91]. The limited nutrients in the soil prompt the succession of fungal community to develop more efficient nutrient utilization efficiency [84], and it is speculated that this may be the reason underlying the more complex fungal network of *Meconopsis integrifolia*. 

The tightness of connections between species (nodes) in a network can be characterized by the mean path distance. Shorter mean path distances imply closer interactions between species in that network. However, it is implied that external disturbances spread faster through the network of more tightly connected communities, which may therefore be more sensitive to disturbances [92]. We found here that the average path length was longer for bacteria than fungi. It is hypothesized that although *Meconopsis integrifolia* soil fungi are more complex than bacteria, they are more sensitive to external disturbances. Network modularity is normally used to characterize the resistance of a system to external disturbances, and the higher the network modularity, the stronger its resistance to external disturbances [93]. The network modularity of bacteria is higher than that of fungi, which also indicates that fungi are less able to resist external disturbances compared with bacteria.

#### 4.2.2. The Identified Networks Are Mostly Positively Correlated 

Positive correlation connections in microbial networks may reflect the presence of synergistic cooperation or overlapping ecological niches between species, while negative correlation connections may imply competitive interactions or ecological niche differentiation [94,95]. The large proportion of positive correlations across the bacterial and fungal networks implies more synergistic and efficient cooperation. It is generally considered that the function of the microbial community depends on the species involved in cooperative metabolism and providing health benefits to the host [96,97]. Although cooperation implies that one species helps another to survive and replicate, thus potentially facilitating colonization, it does not mean stability, as cooperation can create dependence and thus lead to mutual destruction [98]. It has been shown that when faced with extreme harsh environments (for example, intense solar radiation, low temperatures, and soil oxygen levels), the dominance of positive co-occurrence patterns at high altitudes on the Tibetan Plateau suggests the need for potential cooperative interactions between species to survive [88]. *Meconopsis integrifolia* faces a trade-off: although increased intra-community cooperation is expected to promote overall metabolic efficiency, this comes at the cost of reduced ecological stability [99,100,101]. This may hint at the reason for the endangerment of *Meconopsis integrifolia*.

#### 4.2.3. Importance of Keystone Strains

The larger the node, the greater the role in the network, and the largest node is the keystone of the network [102]. Microbial keystone taxa are highly connected taxa. These taxa have unique and critical roles in microbial communities, and their removal may lead to dramatic changes in microbial community structure and function [103]. The key strains in the bacterial network are *unclassified_Caulobacteraceae* and *Pedobacter*, which are found to be in low abundance. A certain strain of *Pedobacter* has been patented as an environmentally friendly microbial agent that decomposes the mycelium of plant pathogens, including fungal pathogens such as *Fusarium*, which consist of titin as cell wall [104]. Fifteen new strains of the genus *Pedobacter* that were isolated from Arctic soils demonstrate optimal grow at 15–20 °C. Genes related to adaptation to cold environments are present in the strains and have potential antioxidant activity [105]. The optimum temperature for the growth of this genus is consistent with the temperature at the site of the present collection of *Meconopsis integrifolia*, and it is speculated that the key microorganisms for sustaining the growth of *Meconopsis integrifolia* must be cold-tolerant. The key strain that sustains the growth of *Meconopsis integrifolia* suppresses pathogenic bacteria, which may indicate that *Meconopsis integrifolia* has high requirements in terms of soil conditions, since soil can contain many disease-causing microorganisms. The fact that there are no companion plants around *Meconopsis integrifolia* may suggest it requires only a simple soil environment for survival. The potential antioxidant activity suggests the possible involvement of this genus in the growth, development, metabolic processes, and accumulation of active ingredients of *Meconopsis integrifolia*. Caulobacteraceae are cold-tolerant and have excellent adaptability to oligotrophic habitats. Some strains produce IAA and promote plant growth [106]. Of the remaining strains with greater connectivity, members of the family *Flammeovirgaceae* are resistant to gentamicin, kanamycin, neomycin, and streptomycin and demonstrate resistance to gentamicin, kanamycin, neomycin, and streptomycin [107]. *Planctomycetaceae* are highly tolerant to low temperatures [108] and found to harbor genes for the biosynthesis of alkaloids [109]. Interestingly, *Cyanobacteria* are uniquely adapted to high-latitude environmental conditions, demonstrating tolerance to high UV radiation and desiccation as well as mechanisms to protect cells from freeze–thaw damage. They are major contributors to vital ecosystem services in the polar regions, particularly carbon and nitrogen conversion in terrestrial polar habitats [110]. It is widely accepted that *Cyanobacteria* are largely responsible for providing the most important ecosystem services. Cyanobacterial autotrophy supports a large diversity of heterotrophic microorganisms (for example, Actinbacteria, Proterobacteria, Firmicutes, and Bacteroidetes) as well as less abundant organisms or organisms at higher trophic levels [111,112,113,114,115,116]. Therefore, it is highly probable that *Cyanobacteria* are responsible for important ecosystem services. Some studies have reported the enrichment of members of the *Chitinophagaceae* and *Flavobacteriaceae* families in plants during pathogenic invasion, which promotes plant growth and tolerance to stress [117].

Compared with bacteria, the key node species of the soil fungal network are more abundant, and most belong to Ascomycetes, Basidiomycota, Rozellomycota, and Mortierellomycota, all four of which have members that are the dominant species in the soil fungal community. Ascomycota accounts for the largest proportion, of which share of the keystone Xylariaceae is associated with a patent that can be used to produce cellulase and bacterial fertilizers that can degrade bioorganic matter [118]. *Helotiales* has plant growth-promoting properties [119]. Isolates of Helotiales fungi from the traditional medicinal plant Bergenia pacumbis have great potential for structurally diverse natural products, including polyketides, terpenoids, etc. [120]. *Fusidium* can be used to prepare antibiotics and has activity against certain pathogenic bacteria [121]. *Preussia* produces IAA for plant growth [122]. The extracts of *Phaeosphaeria* contain polyketides and have antibacterial properties [123]. Some strains of *Coniochaeta* degrade cellulose and produce cellulase, and some strains can be used to promote blood clotting, treat stress and cancer, and lower blood cholesterol [124]. *Myrothecium* can be used as a bioherbicide or biocontrol agent, and the cultured mycelium can produce antibiotics [125,126,127]. *Collophora* is a cold-adapted yeast that reduces or avoids cell damage from UV radiation [128], and the characteristic of this genus corresponds to the high-radiation environment in which *Meconopsis integrifolia* is found. A certain strain of *Duddingtonia* also promotes plant growth and development [129]. *Simplicillium* can be used to prepare plant pathogenic bacterial preparations and plant coatings [130]. A novel antibiotic with antibacterial activity was isolated and purified from a fungus symbiotic with *Cyanobacteria*, and the antibiotic metabolite was found to be a novel antibiotic with a structure similar to alkaloids [131]. *Beauveria bassiana* has biocontrol potential against plant diseases and has a growth-promoting effect on host plants. It can produce flavonoids, alkaloids, and other natural products and shows excellent antitumor, antibacterial, antiviral, and insecticidal activities [132]. *Serendipita* is used for the preparation of inoculum, promoting root colonization of host plants. Some members of *Serendipitaceae* often coexist with clumping mycorrhizal fungi (AMF), and application of williamsii on mycorrhizal tomato plants significantly increases nitrogen concentrations [133]. Almost all species within *Tomentella* are ectomycorrhizal (EcM) fungi [134] and act as major mediators of soil biomes by exchanging soil nutrients with host plants for photosynthetic carbon, which plays an important role in nutrient cycling. They are important for nutrient cycling in host plants and can provide substantial protection to host plants by encapsulating root tips and acidifying the soil [135]. It has been reported in some studies that elevated atmospheric temperatures [136,137] and long-term nitrogen deposition [138,139] lead to a decrease in EcM fungal diversity. This may be the reason why high temperatures at low altitudes are not conducive to the growth of *Meconopsis integrifolia*. Regarding negative correlations, *Paraphaeosphaeria* has antifungal activity [140], and *Cystobasidium* species can grow at subzero temperatures and in vitamin-free media. *Mortierella* can remain active at lower temperatures and has an advantage under unfavorable soil conditions [74]. Its activity in agricultural soils plays an important role in the growth and development of cultivated plants and their resistance to stress [141,142]. The genus *Erysipelas* is one of the largest and most morphologically diverse genera of stretcher mushrooms in the world. They are widely distributed geographically and ecologically and form ectomycorrhizal relationships with a variety of plants [143]. Ectomycorrhizal mycorrhizal fungi (EMFs) are reciprocal symbiotic partners associated with host plants. In this relationship, the fungi help the plant to obtain minerals and water and enhance the plant’s resistance to pathogenic infections and environmental stresses [144,145,146]. In return, host plants provide carbon rates and unique ecological niches for these fungi [147,148]. *Stilbella* microorganisms are able to use flavonoids as a carbon source [149], and strains can be cultured and used to prepare novel physiologically active substances for cancer treatment [150].

It has been shown that plants in microbial networks defend against pathogenic attack by recruiting certain keystone species [151], and key taxa may use a range of strategies to exert influence on the microbiota. For example, they may act through intermediate or effector groups whose abundance can be selectively regulated to modulate community structure and function [152,153]. Such selective regulation may include promoting (commensal) or inhibiting (non-commensal) effector groups through the secretion of metabolites, antibiotics, or toxins [103]. Most of the key species in the fungal network are cold-tolerant and radiation-resistant. They can promote plant root colonization and plant growth, resist disease stress, and are beneficial to *Meconopsis integrifolia* plants, especially in the presence of mycorrhizal fungi, endophytic mycorrhizae, and ectomycorrhizae, all of which indicate the adaptation of soil microorganisms of *Meconopsis integrifolia* growing on the Tibetan Plateau to the environment. It should not be overlooked that most of the key strains produce natural products with antimicrobial or antioxidant activity. Usually, they are part of the ecological defense system of the organism, as a product of stress response against competing life forms or for improved survival in cases of environmental stress [154]. Their presence indirectly plays a protective role in the survival of *Meconopsis integrifolia* plants. If these products could replace the medicinal effects of the *Meconopsis integrifolia* itself, and once this possibility becomes a reality, it could certainly allow reducing the large-scale harvesting of wild *Meconopsis integrifolia* in the future, facilitating conservation of its rare germplasm resources, which would be a remarkable achievement. A small subset of highly connected key microorganisms can be the best predictor of overall community change [155]. High levels of disturbance and dispersion can destabilize the role of keystone taxa, and the removal of key microorganisms can lead to dramatic changes in the composition and function of the microbiome. These key strains are unique and important to *Meconopsis integrifolia*, so if plants did not build up or could not reach the corresponding ecological function within a certain period of arrival in the new environment, this may be one of the reasons why *Meconopsis integrifolia* cannot survive outside its alpine habitat. However, co-occurrence patterns are statistically determined associations between the relative abundances of various OTUs, which can only indicate potential positive, negative, or neutral interactions, and must be interpreted with caution. Future studies can further validate the importance of key taxa by isolating and identifying key taxa from *Meconopsis integrifolia* soil microbes and exploring their function. Its importance can be further verified if the deletion of key strains has negative effects or leads to either dysfunction or even recovery of function.

### 4.3. Potential Growth-Promoting Bacteria in Culturable Microorganisms

Many bacteria related to plant promotion were found in the community composition through high-throughput sequencing, so bacterial isolation and identification were performed, and it was found that many functional microorganisms are present in this community. The abundance of *Actinomycetes* among bacteria isolated from *Meconopsis integrifolia* soil was the highest, and the percentage of *Streptomyces* was the largest, which is consistent with the results of Fan et al. [156], who isolated *Streptomyces* as the dominant genus (78%) in endophytic and inter-rhizosphere isolation of *Meconopsis punicea* using a culture-free method. It is also consistent with the results of Chi et al. that the dominant culturable bacteria of soil in multiple regions of Tibet are *Streptomyces* [157]. *Streptomyces* can protect plants from pathogens and is known for secreting antibiotics, which can be beneficial for *Meconopsis integrifolia* and also provide new avenues for researching new drugs. It is a good resource to reduce overexploitation of wild *Meconopsis*, so subsequent studies can focus on the relationship between actinomycetes and medicinal components of *Meconopsis*. IAA is reported to be involved in the growth regulation of plant roots and stems [158]. Phosphorus-solubilizing microorganisms in soil not only promote plant growth through their own secreted products, but also interact with each other to produce antagonistic effects on other pathogenic bacteria [159,160]. The combined application of phosphorus-solubilizing microbial agents and chemical fertilizers also helps to increase the density of microbial populations and improve the soil microhabitat. The secretion of iron carriers can inhibit the growth of pathogenic bacteria and, in some cases, induce systemic resistance [161]. Therefore, *Pseudomonas laurylsulfatiphila*, *Microbacterium algeriense*, and *Peribacillus simplex*, *Streptomyces murinus*, *Microbacterium phyllosphaerae*, *Streptomyces thermoviolaceus* subsp. *Thermoviolaceus*, and *Streptomyces rishiriensis*, isolated in this study, are found to play a beneficial role in the growth of *Meconopsis integrifolia*. They thus represent important strain resources that can be used in the future production of biofertilizer to regulate the soil microenvironment of *Meconopsis integrifolia*.

## 5. Conclusions

This study is the first high-throughput analysis of the soil microbial community of artificially cultivated *Meconopsis integrifolia* and the mining of pure culture resources. Most of the soil bacteria of *Meconopsis integrifolia* are found to be involved in carbon and nitrogen metabolism, organic matter degradation, and nutrient cycling, while mycorrhizal fungi promote plant growth and alleviate disease stress, thus assuming greater ecological functions than the bacteria. Certain bacteria, especially most fungi, which play an important role in the soil, have anticancer and antiviral activities or can produce functional components such as alkaloids. *Meconopsis integrifolia* favors more cooperation to promote overall metabolic efficiency at the cost of reduced ecological stability, revealing a possible strategy used by *Meconopsis integrifolia* in the face of dilemmas related to survival. The beneficial strains that can be cultivated in this study and the keystones exerted in the network of *Meconopsis integrifolia* are yet to be experimentally verified, but our study can contribute to better understanding how *Meconopsis integrifolia* survives in the plateau environment and provide more useful information on the artificial cultivation of this precious and endangered traditional medicinal plant. The pioneering results are of significance for the conservation of this plant, serving as guidance of future cultivation and even the sustainable exploitation of its medicinal value.

## Figures and Tables

**Table 1:** Diversity index.

Classified	Sample	Number of Sequences	Number of OTUs	ACE	Chao1	Shannon	Simpson
Bacteria	M1	105,298	2759	3049.37	3020.17	6.80	0.002747
	M2	87,264	2756	3059.27	3065.06	6.93	0.002269
	M3	89,772	2469	2826.53	2819.84	6.57	0.003527
	M4	93,596	2612	2939.35	2923.72	6.65	0.004035
	M5	76,373	2784	3076.36	3056.26	6.88	0.002544
	M6	107,230	2840	3098.08	3114.25	6.89	0.002408
	M7	94,746	2877	3147.46	3132.49	6.89	0.002575
	M8	127,350	2873	3190.22	3156.52	6.70	0.003976
Fungi	M1	39,471	319	433.62	435.68	3.81	0.060223
	M2	82,544	590	633.25	644.05	3.72	0.065641
	M3	34,032	478	530.93	518.58	3.98	0.05309
	M4	40,498	492	574.20	571.94	2.83	0.227684
	M5	46,018	509	546.35	557.46	3.83	0.064865
	M6	45,850	483	517.35	522.06	3.53	0.143607
	M7	36,850	474	521.56	513.72	4.11	0.046816
	M8	84,407	552	588.28	588.98	3.78	0.065266

**Table 2:** The characteristics of the co-occurrence networks.

Network Indexes	Bacteria	Fungi
Total nodes	624	627
Total links	479	5330
Average degree	1.535	17.002
Diameter	9	2
Density	0.002	0.027
Average clustering coefficient	0.622	0.967
Average path distance	1.973	1.033
modularity	0.986	0.84

**Table 3:** Qualitative and quantitative determination of N1-12, N1-21, and N1-73.

Measuring Item	CK (Sterile Water)	CK (IAA)	N1-12	N1-21	N1-73
Qualitative color	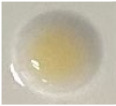	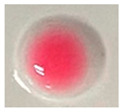	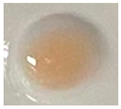	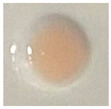	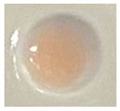
Quantitative determination (IAA μg/mL)	0.00	20	1.57	2.82	3.84

**Table 4:** Average organic phosphorus solubilization rate, inorganic phosphorus solubilization rate, and iron production carrier rate for all bacteria.

Genus	Strain Name	Average Dissolved Organic Phosphorus Rate (D/d)	Average Iron Production Carrier Rate (D/d)	Average Dissolved Inorganic Phosphorus Rate (D/d)
*Streptomyces*	N1-26	1.64	1.64	-
	R2-10	2.06	1.28	-
	R2-13	1.72	-	-
	R2-18	1.69	1.63	-
*Peribacillus*	N1-13	1.27	-	-
	N1-73	1.95	1.82	-
*Ensifer*	N1-6	1.71	1.74	-
*Bacillus*	N1-18	1.24	-	-
*Pedobacter*	N1-17	1.61	-	-
*Flavobacterium*	N1-4	1.49	-	-
*Pseudomonas*	N1-12	1.36	1.36	1.37
*Microbacterium*	N1-72	1.75	2.14	-
	N1-21	-	1.24	-
	N1-15	-	1.24	-

## Data Availability

The data presented in this study are available in the Supplement Material.

## Supplementary Materials

The following supporting information can be downloaded at https://www.mdpi.com/article/10.3390/biology12020160/s1. Table S1: Total culturable bacteria. Table S2: IAA standard curve. Table S3: Average organic phosphorus solubilization rate, inorganic phosphorus solubilization rate, and iron production carrier rate for all bacteria.
